# Kappa free light chain index predicts long-term disease activity and disability accrual in multiple sclerosis

**DOI:** 10.1177/13524585251344807

**Published:** 2025-06-16

**Authors:** Klaus Berek, Martin Schmidauer, Gabriel Bsteh, Michael Auer, Robert Barket, Thomas Berger, Franziska Di Pauli, Astrid Ellen Grams, Michaela Hassler, Lukas Lenhart, Dejan Milosavljevic, Anne Zinganell, Janette Walde, Florian Deisenhammer, Harald Hegen

**Affiliations:** Department of Neurology, Medical University of Innsbruck, Innsbruck, Austria; Department of Neurology, Medical University of Innsbruck, Innsbruck, Austria; Department of Neurology, Medical University of Vienna, Vienna, Austria; Comprehensive Center for Clinical Neurosciences and Mental Health, Medical University of Vienna, Vienna, Austria; Department of Neurology, Medical University of Innsbruck, Innsbruck, Austria; Department of Neurology, Medical University of Innsbruck, Innsbruck, Austria; Department of Neurology, Medical University of Vienna, Vienna, Austria; Comprehensive Center for Clinical Neurosciences and Mental Health, Medical University of Vienna, Vienna, Austria; Department of Neurology, Medical University of Innsbruck, Innsbruck, Austria; Department of Radiology, Medical University of Innsbruck, Innsbruck, Austria; Neuroimaging Research Core Facility, Medical University of Innsbruck, Innsbruck, Austria; FH Campus Wien, University of Applied Sciences, Vienna, Austria; Department of Radiology, Medical University of Innsbruck, Innsbruck, Austria; Neuroimaging Research Core Facility, Medical University of Innsbruck, Innsbruck, Austria; FH Campus Wien, University of Applied Sciences, Vienna, Austria; Department of Neurology, Medical University of Innsbruck, Innsbruck, Austria; Department of Statistics, Faculty of Economics and Statistics, University of Innsbruck, Innsbruck, Austria; Department of Neurology, Medical University of Innsbruck, Innsbruck, Austria; Department of Neurology, Medical University of Innsbruck, Innsbruck, Austria

**Keywords:** Multiple sclerosis, kappa free light chain, prognosis, long-term, outcome

## Abstract

**Background:**

The prognostic value of κ-free light chain (κ-FLC) index over the long term is unknown.

**Objectives:**

The objective of the study was to determine whether κ-FLC index determined at disease onset predicts relapse activity and disability accrual during long-term follow-up.

**Methods:**

Patients with a first demyelinating event of the central nervous system who had cerebrospinal fluid and serum sampling were eligible for inclusion. At baseline, demographics, clinical data, number of T2 hyperintense (T2L) and contrast-enhancing lesions (CEL) on MRI were assessed. During follow-up occurrence of relapses, Expanded Disability Status Scale (EDSS) scores and disease-modifying treatments (DMT) were registered. κ-FLC was measured by nephelometry and κ-FLC index calculated as (CSF κ-FLC/serum κ-FLC)/albumin quotient.

**Results:**

Sixty-four patients with a median age at onset of 32 years (25th−75th percentile: 27−39) and a female predominance of 75% were followed over a median of 113 (90−129) months. Forty-six (72%) patients experienced relapse, 30 (47%) showed disability accrual. Multivariable Cox regression analysis adjusted for age, sex, disease duration, T2L, CEL and DMT revealed that κ-FLC index independently predicts time to relapse (per increase of 10: hazard ratio (HR) = 1.04, lower limit (LL)-confidence interval (CI) = 1.001, *p* = 0.044) and disability accrual (per increase of 10: HR = 1.05, LL-CI = 1.009, *p* = 0.022).

**Conclusions:**

κ-FLC index predicts long-term disease activity independently of other risk factors.

## Introduction

Multiple Sclerosis (MS) is a chronic inflammatory immune-mediated disease of the central nervous system (CNS) and the most frequent cause of permanent disability in young adults.^1^ The course of MS shows a large inter-individual heterogeneity,^2^ thus, weighing benefits and risks of available immune treatments has become a key challenge for neurologists counselling patients with MS.^3^ Consequently, biomarkers aiding reliable prognosis at the time of diagnosis are urgently needed.

κ-free light chain (κ-FLC) index is an emerging body fluid marker in MS, which is supposed to contribute to personalized MS treatment.^4^ κ-FLC index reflects intrathecal B cell activity, shows a high diagnostic accuracy in MS and predicts early MS disease activity.^5^ Higher κ-FLC index determined at the time of disease onset was associated with various outcome measures in MS, including shorter time to relapse,^6^ new magnetic resonance imaging (MRI) activity,^7^ disability accrual^8^ as well as with a shorter time to cognitive decline.^9^ While most of the studies followed patients only over a few years, there is a lack of evidence regarding the predictive value of κ-FLC index over the long term.

Hence, the aim of the present study was to investigate whether κ-FLC index determined at disease onset predicts relapse activity and disability accrual during long-term follow-up.

## Methods

### Study design and patients

From an ongoing observational study^6^ initiated in 2007, of patients with a first demyelinating event of the CNS suggestive of MS at the MS clinic of the Medical University of Innsbruck who (1) had the diagnosis of clinically isolated syndrome (CIS) or relapsing-remitting MS (RRMS) according to the McDonald criteria 2017,^10^ and (2) received cerebrospinal fluid (CSF) and serum sampling within 6 months after symptom onset, we included those with a clinical follow-up of at least 5 up to 15 years. For further details of the original cohort, we refer to the previous publication.^6^

Briefly, demographic data (age, sex), clinical (disease duration) and imaging characteristics (number of hyperintense lesions on T2-weighted MRI (T2L), number of contrast-enhancing lesions (CEL)) were assessed at baseline. Disease duration was defined as time between symptom onset and lumbar puncture.

During follow-up visits, performed twice a year, the occurrence of relapses as well as of disability accrual, assessed by the Expanded Disability Status Scale (EDSS) score,^11^ were recorded. Furthermore, the initiation of disease-modifying therapy (DMT) was registered.

### κ-FLC detection

κ-FLC was determined as previously described.^6^ Briefly, κ-FLC concentrations were measured in CSF and serum by nephelometry (using the N Latex FLC kappa assay and the Behring ProSpec, Siemens, Erlangen, Germany). The intrathecal κ-FLC synthesis was determined by the κ-FLC index:^12^

$$\kappa -FLCindex=\frac{\kappa -{FLC}_{CSF}/\kappa -{FLC}_{Serum}}{{Albumin}_{CSF}/{Albumin}_{Serum}}$$

A κ-FLC index ⩾6.1 denoted the presence of an intrathecal κ-FLC synthesis and was termed as ‘positive’, while an index below this cut-off was considered as ‘negative’.^13^ A κ-FLC index >100 was defined as ‘high’ κ-FLC index.^6^

### Research question

Does κ-FLC index determined at the time of first disease manifestation predict relapse activity and disability accrual in MS patients during long-term follow-up?

### Endpoints

The primary endpoint was time to relapse. The secondary endpoint was time to disability accrual.

### Definitions of clinical endpoints

A relapse was defined as patient-reported symptoms confirmed by a neurologist or objectively observable clinical signs typical of an acute inflammatory demyelinating event in the CNS with a duration of at least 24 hours. Relapses had to occur in the absence of fever or infection and had to be separated from previous relapses by at least 30 days.^14^

Disability accrual was defined as an EDSS score increase of ⩾1.5 in case of an EDSS baseline score of 0, of ⩾1.0 in case of EDSS baseline scores ⩾1.0 and ⩽5.5, or ⩾0.5 in case of EDSS baseline scores of >5.5 and had to be confirmed after at least 12 months.^15^

### Statistical analysis

Statistical analysis was performed using R.^16^ Data were displayed as frequencies (percentages) and median (25th–75th percentile) as appropriate. For group comparisons Mann–Whitney *U* test or χ^2^ test were applied.

To identify predictors of the primary endpoint time to relapse (dependent variable), multivariable Cox regression was performed using κ-FLC index (continuous) as the independent variable adjusting for age (continuous), sex (binary), disease duration (continuous), T2L (continuous), CEL (continuous) and DMT administration (binary).^6^ Similarly, Cox regression was employed with time to disability accrual (secondary endpoint) as dependent variable.

A *p* value <0.05 was considered statistically significant. One-sided hypothesis testing was used based on clear pre-specified 1-sided hypotheses. Prior studies provide the evidence on the direction of the effects of the independent variables (used in the Cox regression model) on the dependent variable (endpoints), that is, an increased risk of relapse by higher MRI activity (higher T2L^17^ and CEL^18^), younger age,^17^ female sex,^19^ shorter disease duration,^20^ no DMT use^21–25^ and higher κ-FLC index,^6,7^ as well as an increased risk of disability accrual by higher MRI activity (higher T2L^26^ and CEL^26^), higher age,^27^ male sex,^19^ longer disease duration,^28^ no DMT use^21–25^ and higher κ-FLC index.^6,8,17^ Thus, 1-sided *p* value and 1-sided 95% confidence interval (CI), that is, either the lower limit (LL) or upper limit (UL), were shown.

To visualize the effect of the κ-FLC index, we computed the estimated Cox regression survival probabilities separately for high and low κ-FLC index values. κ-FLC index values >100 were considered as high, low κ-FLC index values were set ⩽100. The median of these high and low κ-FLC index values, respectively, was used to plug into the Cox regression to compute the graph. In addition, DMT was set to ‘no treatment’, sex to ‘female’, and all other parameters were set to their median values.

### Ethics

The study was approved by the ethics committee of the Medical University of Innsbruck (approval number: 1249/2023). Written informed consent was obtained from all patients. We adhered to the declaration of Helsinki and national regulations during all study procedures.

## Results

Of the initial 88 patients,^6^ 64 (73%) had long-term follow-up over a median period of 113 (90−129) months and were included into the study. Patients were predominantly female (75%), had a median age at baseline of 32 (27−39) years and a median disease duration of 0.4 (0.2−1.3) months. Baseline MRI showed a median of 10 (5−18) T2L and 1 (0−2) CEL (Table 1). κ-FLC index at baseline had a median of 40.2 (19.0−103.3). While the κ-FLC index was positive in 60 (94%) patients, it reached high values (>100) in 17 (27%) cases. There were no relevant differences between the actual and the original cohort (Supplemental Table 1).

**Table 1.** Demographic, clinical and paraclinical characteristics.

Number of patients	64
Baseline characteristics	
Sex (female)	48 (75)
Age (years)	32 (27–39)
Disease duration (months)^a^	0.4 (0.2−1.3)
Diagnosis at baseline^b^	
Clinically isolated syndrome	12 (19)
Relapsing remitting MS	52 (81)
EDSS	0 (0−1)
Number of T2L	10 (5−18)
Number of CEL	1 (0−2)
OCB positive	61 (95)
Follow-up characteristics	
Follow-up duration (months)^c^	113 (90−129)
Relapse activity	46 (72)
DMT administration before relapse^d^	19 (30)
Disability accrual	30 (47)
DMT administration before disability accrual^e^	37 (58)

Data are shown as median (25th−75th percentile) and *n* (%), as appropriate. CEL: contrast-enhancing lesions on T1-weighted MRI, DMF: dimethyl fumarate, DMT: disease-modifying treatment, EDSS: Expanded Disability Status Scale, GLAT: glatiramer acetate, IFN: interferon-beta, MRI: magnetic resonance imaging, MS: Multiple Sclerosis, NTZ: natalizumab, OCB: oligoclonal bands, OFA: ofatumumab, RTX: rituximab, S1P: sphingosine-1-phosphate receptor modulator, TER: teriflunomide, T2L: hyperintense lesions on T2-weighted MRI.

a Is defined as time from symptom onset to lumbar puncture.

b According to the 2017 revised McDonald criteria.

c Duration from baseline to last visit.

d DMT start before relapse or until the end of follow-up in non-relapsing patients (5 DMF, 3 GLAT, 2 IFN, 2 TER, 1 RTX, 1 consecutively given GLAT/DMF, 1 IFN/GLAT, 1 TER/DMF, 1 GLAT/NTZ, 1 IFN/NTZ, 1 IFN/S1P).

e DMT start before disability accrual or until the end of follow-up in stable patients (7 DMF, 2 GLAT, 11 IFN, 2 TER, 1 RTX, 1 consecutively given GLAT/DMF, 3 GLAT/IFN, 1 TER/DMF, 3 IFN/NTZ, 1 GLAT/NTZ, 2 IFN/S1P, 2 GLAT/S1P, 1 DMF/OFA).

During the observation period, a total of 46 patients (72%) had at least one relapse. Nineteen (30%) patients received DMT prior to relapse or until the end of follow-up in non-relapsing patients. Thirty (47%) patients suffered disability accrual during follow-up. Thirty-seven (58%) received DMT before disability accrual or until the end of follow-up in stable patients. For details see Table 1.

### κ-FLC index is associated with shorter time to relapse activity

Patients with relapse activity during follow-up were younger (30 (26−37) years) and showed a significantly higher number of T2L (11 (8−20)) and CEL (2 (0−2)) at baseline compared to patients without relapse activity (38 (28−41), *p* = 0.047; 3 (0−8), *p* = 0.003 and 0 (0−1), *p* = 0.020).

κ-FLC index was higher in patients with a relapse (41 (22−125)) than in patients without relapse activity (34 (13−57), *p* = 0.029). Results of univariate testing are provided in Supplemental Table 2.

**Figure 1.**
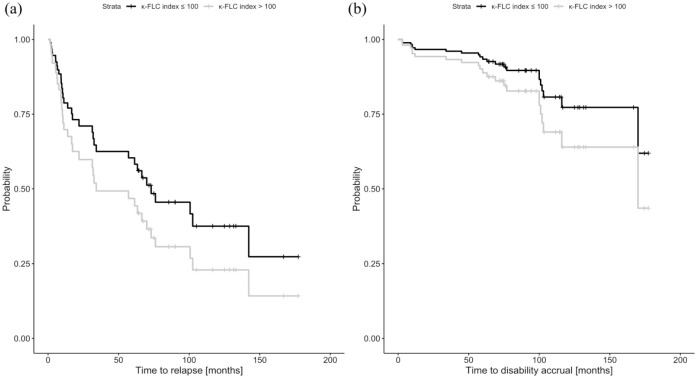
Probability of (a) relapse and (b) disability accrual depending on baseline κ-FLC index. The probability of developing (a) relapse or (b) disability accrual during 10-year follow-up was higher in the high κ-FLC index (>100) group (*n* = 17) than in the low κ-FLC index (⩽100) group (*n* = 47; *p* = 0.049 and 0.008). For computing the estimated survival probabilities, the median of high (147) and low κ-FLC index (29) values, respectively, was used. In addition, DMT was set to ‘no treatment’, sex to ‘female’, and all other parameters were set to their median values (multivariable Cox regression). κ-FLC: κ-free light chain.

Multivariable Cox regression analysis revealed κ-FLC index as an independent predictor of time to relapse activity (per increase of 10: hazard ratio (HR) = 1.04, LL-CI = 1.001, *p* = 0.044, Table 2). Patients with high κ-FLC index at baseline (>100, median value 147) displayed a significantly shorter median time to relapse with 34 months compared to 73 months in those with low κ-FLC index (⩽100, median value 29; Figure 1(a)). At year 10, the high κ-FLC index group had a 39% decreased probability to remain free of relapse compared to patients with a low κ-FLC index.

**Table 2.** Cox regression analyses for prediction of time to relapse (A) and to disability accrual (B).

A	Relapse activity					
	Coefficient	Standard Error	HR	LL-CI	UL-CI	*p* value
**κ-FLC index**	0.003	0.002	1.035*	1.001*	–	**0.044**
**Age** (years)	−0.038	0.022	0.962	–	0.998	**0.043**
**Sex** (ref: male)	0.191	0.405	1.211	–	–	0.318
**T2L number**	0.009	0.009	1.009	–	–	0.158
**CEL number**	0.249	0.111	1.282	1.068	–	**0.013**
**DMT administration** (ref: no)	−0.009	0.428	0.991	–	–	0.492
**Disease duration** (months)	0.065	0.090	1.067	–	–	0.236
Cox & Snell *R*^2^	0.36					
B	Disability accrual					
	Coefficient	Standard error	HR	LL-CI	UL-CI	*p* value
**κ-FLC index**	0.005	0.002	1.048*	1.009*	–	**0.022**
**Age** (years)	−0.001	0.026	0.999	–	–	0.479
**Sex** (ref: male)	−0.902	0.496	0.406	–	0.917	**0.034**
**T2L number**	−0.003	0.013	0.997	–	–	0.418
**CEL number**	0.043	0.136	1.044	–	–	0.375
**DMT administration** (ref: no)	0.832	0.560	2.297	–	–	0.069
**Disease duration** (months)	0.082	0.093	1.086	–	–	0.188
Cox & Snell *R*^2^	0.40					

Cox regression models with time to relapse (A) and time to disability accrual (B) as dependent variables. One-sided *p* values <0.05 were considered statistically significant and marked bold. Age, T2L and CEL were determined at the time of lumbar puncture. DMT administration was determined until occurrence of relapse/ disability accrual or end of observation, respectively. CEL: contrast-enhancing lesions on T1-weighted MRI; CI: confidence interval; DMT: disease-modifying treatment; FLC: free light chain; HR: hazard ratio; MRI: magnetic resonance imaging; LL: lower limit. ref: reference category; T2L: hyperintense lesions on T2-weighted MRI; UL: upper limit.

* Per increase of 10.

### κ-FLC index is associated with shorter time to disability accrual

Patients with disability accrual during follow-up showed a significantly higher number of CEL (2 (1−2)) and a higher κ-FLC index (80 (33−147)) at baseline compared to patients without disability accrual (0 (0−2), *p* = 0.042; 29 (16−51), *p* = 0.002). Results of univariate testing are provided in Supplemental Table 2.

Cox regression analysis revealed κ-FLC index also as an independent predictor of the time to disability accrual (per increase of 10: HR = 1.05, LL-CI = 1.009, *p* = 0.022, Table 2). Patients with high κ-FLC index at baseline showed disability accrual in 36% of cases after 10 years, whereas patients with low κ-FLC index in 23% of cases (Figure 1(b)). The estimated chance to remain without disability accrual at year 10 in patients with high κ-FLC index was decreased by 17% compared to patients with low κ-FLC index.

## Discussion

In this study, we investigated the prognostic value of κ-FLC index over the long term in patients with MS. We observed that κ-FLC index determined at disease onset predicts time to relapse and time to disability accrual independently of other well-known prognostic factors including MRI activity (e.g. T2L) or DMT administration over approximately 10 years.

MS is characterized by varying degrees of inflammation, demyelination and neurodegeneration within the CNS.^1^ Due to its heterogeneous clinical disease course predicting future relapse activity and disability accrual is still a challenge in patient management. While κ-FLC index has demonstrated its potential to predict disease activity^5,6^ over the short term, data over the long term and especially with regard to prediction of disability accrual are scarce. To date, only one study has examined the performance of κ-FLC index levels for long-term prognostication in MS.^29^ Herein, no statistically significant differences of κ-FLC index values in patients with benign MS (defined as EDSS values of ⩽3 after 10 years) compared to patients with aggressive MS (defined as EDSS values of ⩾6 after 15 years) were reported. However, this negative finding might be explained by the low sample size of the study (*n* = 35) and, thus, insufficient statistical power.

In a clinical setting, the prognostic value of the κ-FLC index may assist in tailoring therapeutic interventions. It has to be embedded in the current discussion of early highly effective DMT application.^30,31^ Probably, there is no simple ‘one-fits-all’ approach with regard of DMT management in MS, but on the contrary a more personalized approach is needed.^32^ κ-FLC index may help to stratify MS patients at disease onset according to their risk of future relapse activity and disability accrual. High κ-FLC index – together with other risk factors such as incomplete recovery from the first clinical attack or high number of MRI lesions^26,27^ – might trigger early highly effective DMT. There is overwhelming evidence that early treatment significantly delays conversion to clinically definite MS (CDMS) as well as disability progression.^33^ Conversely, in patients who probably will show a mild disease course a potentially harmful, psychologically distressing, and, last but not least, costly DMT is not needed.^34^

In terms of its routine application, κ-FLC index as a fast, economic and reliable method^35^ and it is supposed to be integrated into the next revision of MS diagnostic criteria as alternative to oligoclonal banding.^12^ This means that κ-FLC index will be widely applicable and can be easily used for risk assessment.

There are some limitations to this study. First, we could include only 64 of the initial 88 patients into the present analysis due to loss of follow-up. The patients in the present study show similar characteristics at baseline compared to the original cohort (Supplemental Table 1), but we cannot exclude some effects due to selection bias. Second, although we achieved statistically significant hazard ratios for the κ-FLC index, the number of patients and the number of events (i.e. relapses and disability accrual, respectively, during follow-up) were still small. This means that results that are not statistically significant (e.g. of MRI parameters) might still have an impact. In addition, we could not adjust our models for corticosteroid treatment before lumbar puncture and storage time until measurement. However, these two factors most probably do not impact our findings as previously investigated.^6,36^ Cox regression models adjusted for baseline EDSS are given in Supplemental Table 3 showing overall similar results. We adjusted our models for DMT administration, but we were not able to consider different DMT categories (i.e. no treatment, moderate-efficacy DMT, high-efficacy DMT) due to the small number of patients. We had a relatively low number of patients (approximately one-third) on DMT before the occurrence of relapse. This might be explained by the fact that at the time of disease onset (ranging between the years 2007 and 2016) and treatment decision making, different MS diagnostic criteria were applied. Thus, retrospectively, only 48% of patients received diagnosis of MS directly at disease onset according to the then valid diagnostic criteria. With regard to MRI metrics, we could not consider spinal MRI, as it was not regularly performed and thus not available in a sufficient number of patients. We also have to acknowledge that other endpoints, for example, radiological activity (i.e. new or enlarging T2L or CEL on follow-up MRI scans) or progression independent of relapse activity (PIRA)^33^ would be of interest and provide additional insights. In our cohort, follow-up MRI within similar time intervals was only available in a limited number of patients. Also, PIRA^37^ occurred in only 5 (8%) patients. Thus, follow-up MRI metrics and PIRA could not be used as endpoints in our models, however, are of course intriguing directions of future research. In any case, we provide univariate comparisons of κ-FLC index between patients with and without PIRA in Supplemental Figure 1. Finally, lesion topography was not accessible in our cohort. However, its influence especially on disability accrual would be a further interesting future research direction.

Altogether, in our present study we provide evidence that κ-FLC index predicts also long-term outcome in patients with MS independently of other known prognostic factors. Further studies in a multicenter setting including a higher number of patients are required to replicate our findings.

## Supplemental Material

sj-docx-1-msj-10.1177_13524585251344807 – Supplemental material for Kappa free light chain index predicts long-term disease activity and disability accrual in multiple sclerosis Supplemental material, sj-docx-1-msj-10.1177_13524585251344807 for Kappa free light chain index predicts long-term disease activity and disability accrual in multiple sclerosis by Klaus Berek, Martin Schmidauer, Gabriel Bsteh, Michael Auer, Robert Barket, Thomas Berger, Franziska Di Pauli, Astrid Ellen Grams, Michaela Hassler, Lukas Lenhart, Dejan Milosavljevic, Anne Zinganell, Janette Walde, Florian Deisenhammer and Harald Hegen in Multiple Sclerosis Journal

sj-docx-2-msj-10.1177_13524585251344807 – Supplemental material for Kappa free light chain index predicts long-term disease activity and disability accrual in multiple sclerosis Supplemental material, sj-docx-2-msj-10.1177_13524585251344807 for Kappa free light chain index predicts long-term disease activity and disability accrual in multiple sclerosis by Klaus Berek, Martin Schmidauer, Gabriel Bsteh, Michael Auer, Robert Barket, Thomas Berger, Franziska Di Pauli, Astrid Ellen Grams, Michaela Hassler, Lukas Lenhart, Dejan Milosavljevic, Anne Zinganell, Janette Walde, Florian Deisenhammer and Harald Hegen in Multiple Sclerosis Journal

sj-docx-3-msj-10.1177_13524585251344807 – Supplemental material for Kappa free light chain index predicts long-term disease activity and disability accrual in multiple sclerosis Supplemental material, sj-docx-3-msj-10.1177_13524585251344807 for Kappa free light chain index predicts long-term disease activity and disability accrual in multiple sclerosis by Klaus Berek, Martin Schmidauer, Gabriel Bsteh, Michael Auer, Robert Barket, Thomas Berger, Franziska Di Pauli, Astrid Ellen Grams, Michaela Hassler, Lukas Lenhart, Dejan Milosavljevic, Anne Zinganell, Janette Walde, Florian Deisenhammer and Harald Hegen in Multiple Sclerosis Journal

sj-pdf-4-msj-10.1177_13524585251344807 – Supplemental material for Kappa free light chain index predicts long-term disease activity and disability accrual in multiple sclerosis Supplemental material, sj-pdf-4-msj-10.1177_13524585251344807 for Kappa free light chain index predicts long-term disease activity and disability accrual in multiple sclerosis by Klaus Berek, Martin Schmidauer, Gabriel Bsteh, Michael Auer, Robert Barket, Thomas Berger, Franziska Di Pauli, Astrid Ellen Grams, Michaela Hassler, Lukas Lenhart, Dejan Milosavljevic, Anne Zinganell, Janette Walde, Florian Deisenhammer and Harald Hegen in Multiple Sclerosis Journal
