# Supplementary material for: Kappa free light chain index predicts long-term disease activity and disability accrual in multiple sclerosis
Source: Mult Scler. 2025 Jun 16;31(10):1187–94. doi: 10.1177/13524585251344807 (PMC12432279; doi:10.1177/13524585251344807)
Supplement: sj-docx-1-msj-10.1177_13524585251344807 – Supplemental material for Kappa free light chain index predicts long-term disease activity and disability accrual in multiple sclerosis [file sj-docx-1-msj-10.1177_13524585251344807.docx]

**Supplemental Table 1.** Comparison of demographics and baseline characteristics between the actual study population and the original cohort

|  | 10-Year FU Cohort | Original cohort | p-value^a^ |
| --- | --- | --- | --- |
| Number | 64 | 88 | n.a. |
| Sex (female) | 48 (75) | 60 (68) | 0.315 |
| Age (years) | 32 (27-39) | 33 (26-40) | 0.823 |
| Disease duration (months)^b^ | 0.4 (0.2-1.3) | 0.4 (0.2-1.1) | 0.947 |
| κ-FLC index | 40.2 (19.0-103.3) | 36.5 (15.6-85.0) | 0.498 |
| T2L number | 10 (5-18) | 10 (3-15) | 0.682 |
| CEL number | 1 (0-2) | 1 (0-2) | 0.715 |
| OCB positivity | 61 (95) | 79 (90) | 0.197 |
| DMT administration^c^ | 19 (30) | 20 (23) | 0.811 |

Legend:

Data are shown as median (25^th^-75^th^ percentile) and n (%), as appropriate.

^a^ Two-sided.

^b^ Is defined as time from symptom onset to lumbar puncture.

^c^ Number of patients with DMT start before relapse or until the end of follow-up.

*Abbreviations*: CEL = contrast-enhancing lesions on T1-weighted MRI, DMT = disease-modifying treatment, FU = follow-up, κ-FLC = κ-free light chain, MRI = magnetic resonance imaging, T2L = hyperintense lesions on T2-weighted MRI.
