# Supplementary material for: Kappa free light chain index predicts long-term disease activity and disability accrual in multiple sclerosis
Source: Mult Scler. 2025 Jun 16;31(10):1187–94. doi: 10.1177/13524585251344807 (PMC12432279; doi:10.1177/13524585251344807)
Supplement: sj-docx-2-msj-10.1177_13524585251344807 – Supplemental material for Kappa free light chain index predicts long-term disease activity and disability accrual in multiple sclerosis [file sj-docx-2-msj-10.1177_13524585251344807.docx]

**Supplemental Table 2: Demographic, clinical and paraclinical characteristics according to relapse activity and disability accrual**

|  | **Relapse activity** | | | **Disability accrual** | | |
| --- | --- | --- | --- | --- | --- | --- |
|  | **No**  **(n=18)** | **Yes**  **(n=46)** | **p-value** | **No**  **(n=34)** | **Yes**  **(n=30)** | **p-value** |
| **Age (years)** | 38  (28-41) | 30  (26-37) | **0.047 ^1^** | 32  (27-39) | 31  (26-40) | 0.310 ^1^ |
| **Sex (female)** | 12 (67) | 36  (78) | 0.336 ^2^ | 26  (77) | 22  (73) | 0.772 ^2^ |
| **Disease duration (months)^a^** | 0.5  (0.2-0.9) | 0.3  (0.1-1.4) | 0.138 ^1^ | 0.4  (0.1-1.3) | 0.4  (0.2-1.4) | 0.331 ^1^ |
| **EDSS at baseline** | 1  (0-1) | 0  (0-1) | 0.555 ^1^ | 1  (0-2) | 0  (0-1) | 0.155 ^1^ |
| **κ-FLC index** | 34  (13-57) | 41  (22-125) | **0.029 ^1^** | 29  (16-51) | 80  (33-147) | **0.002 ^1^** |
| **T2L number** | 3  (0-8) | 11  (8-20) | **0.003 ^1^** | 10  (2-20) | 10  (7-15) | 0.203 ^1^ |
| **CEL number** | 0  (0-1) | 2  (0-2) | **0.020 ^1^** | 0  (0-2) | 2  (1-2) | **0.042 ^1^** |
| **DMT administration^b^** | 5  (28) | 14  (30) | 0.834 ^2^ | 16  (47) | 21  (70) | 0.064 ^2^ |

Data are given as median (25^th^-75^th^ percentile) and n (%). Group comparisons performed by ^1^ Mann-Whitney U (one-sided p values <0.05 are marked bold) or ^2^ Chi-square test.

^a^ Is defined as time from time of symptom onset to lumbar puncture.

^b^ DMT start before relapse or until the end of follow-up in non-relapsing patients (5 DMF, 3 GLAT, 2 IFN, 2 TER, 1 RTX, 1 consecutively given GLAT/DMF, 1 IFN/GLAT, 1 TER/DMF, 1 GLAT/NTZ, 1 IFN/NTZ, 1 IFN/S1P). DMT start before disability accrual or until the end of follow-up in stable patients (7 DMF, 2 GLAT, 11 IFN, 2 TER, 1 RTX, 1 consecutively given GLAT/DMF, 3 GLAT/IFN, 1 TER/DMF, 3 IFN/NTZ, 1 GLAT/NTZ, 2 IFN/S1P, 2 GLAT/S1P, 1 DMF/OFA).

*Abbreviations*: CEL = contrast-enhancing lesions on T1-weighted MRI, DMF = dimethyl fumarate, DMT = disease-modifying treatment, EDSS = Expanded disability status scale, GLAT = glatiramer acetate, IFN = interferon, MRI = magnetic resonance imaging, NTZ = natalizumab, OFA = ofatumumab, RTX = rituximab, S1P = Sphingosine-1-phosphate, TER = teriflunomide, T2L = hyperintense lesions on T2-weighted MRI.
