# Supplementary material for: Kappa free light chain index predicts long-term disease activity and disability accrual in multiple sclerosis
Source: Mult Scler. 2025 Jun 16;31(10):1187–94. doi: 10.1177/13524585251344807 (PMC12432279; doi:10.1177/13524585251344807)
Supplement: sj-docx-3-msj-10.1177_13524585251344807 – Supplemental material for Kappa free light chain index predicts long-term disease activity and disability accrual in multiple sclerosis [file sj-docx-3-msj-10.1177_13524585251344807.docx]

**Supplemental Table 3: Cox regression analyses for prediction of time to relapse (A) and to disability accrual (B) with additional adjustment for baseline EDSS**

| **A** | **Relapse activity** | | | | | |
| --- | --- | --- | --- | --- | --- | --- |
|  | **Coefficient** | **Standard Error** | **HR** | **LL-CI** | **UL-CI** | **p-value** |
| **κ-FLC index** | 0.004 | 0.002 | 1.043^*^ | 1.008^*^ | - | **0.022** |
| **Age**  (years) | -0.054 | 0.027 | 0.947 | - | 0.991 | **0.023** |
| **Sex**  (ref: male) | 0.162 | 0.404 | 1.176 | - | - | 0.344 |
| **T2L number** | 0.010 | 0.009 | 1.010 | - | - | 0.132 |
| **CEL number** | 0.253 | 0.107 | 1.288 | 1.081 | - | **0.009** |
| **DMT administration** (ref: no) | 0.043 | 0.439 | 1.044 | - | - | 0.461 |
| **Disease duration** (months) | 0.054 | 0.090 | 1.056 | - | - | 0.274 |
| **Baseline EDSS** | 0.340 | 0.279 | 1.405 | - | - | 0.112 |
| Cox & Snell R^2^ | 0.39 | | | | | |

| **B** | **Disability accrual** | | | | | |
| --- | --- | --- | --- | --- | --- | --- |
|  | **Coefficient** | **Standard Error** | **HR** | **LL-CI** | **UL-CI** | **p-value** |
| **κ-FLC index** | 0.004 | 0.002 | 1.045^*^ | 1.009^*^ | - | **0.034** |
| **Age**  (years) | 0.016 | 0.031 | 1.016 | - | - | 0.298 |
| **Sex**  (ref: male) | -0.926 | 0.489 | 0.396 | - | 0.917 | **0.029** |
| **T2L number** | -0.004 | 0.013 | 0.996 | - | - | 0.374 |
| **CEL number** | -0.010 | 0.154 | 0.990 | - | - | 0.475 |
| **DMT administration** (ref: no) | 0.961 | 0.581 | 2.615 | - | - | 0.050 |
| **Disease duration** (months) | 0.082 | 0.097 | 1.086 | - | - | 0.198 |
| **Baseline EDSS** | -0.374 | 0.348 | 0.688 | - | - | 0.141 |
| Cox & Snell R^2^ | 0.43 | | | | | |

Legend:

Cox regression models with time to relapse (A) and time to disability acrrual (B) as dependent variables. One-sided p values <0.05 were considered statistically significant and marked bold. Age, T2L and CEL were determined at the time of lumbar puncture. DMT administration was determined until occurrence of relapse/ disability accrual or end of observation, respectively. ^*^ per increase of 10

*Abbreviations:* CEL = contrast-enhancing lesions on T1-weighted MRI. CI = confidence interval. DMT= disease-modifying treatment. FLC = free light chain. HR = hazard ratio. MRI = magnetic resonance imaging. LL = lower limit. ref = reference category. T2L = hyperintense lesions on T2-weighted MRI. UL = upper limit.
