# Supplementary material for: Kappa free light chain index predicts long-term disease activity and disability accrual in multiple sclerosis
Source: Mult Scler. 2025 Jun 16;31(10):1187–94. doi: 10.1177/13524585251344807 (PMC12432279; doi:10.1177/13524585251344807)
Supplement: sj-pdf-4-msj-10.1177_13524585251344807 – Supplemental material for Kappa free light chain index predicts long-term disease activity and disability accrual in multiple sclerosis [file sj-pdf-4-msj-10.1177_13524585251344807.pdf]

Supplemental Figure 1: Increased κ-FLC index in patients with PIRA

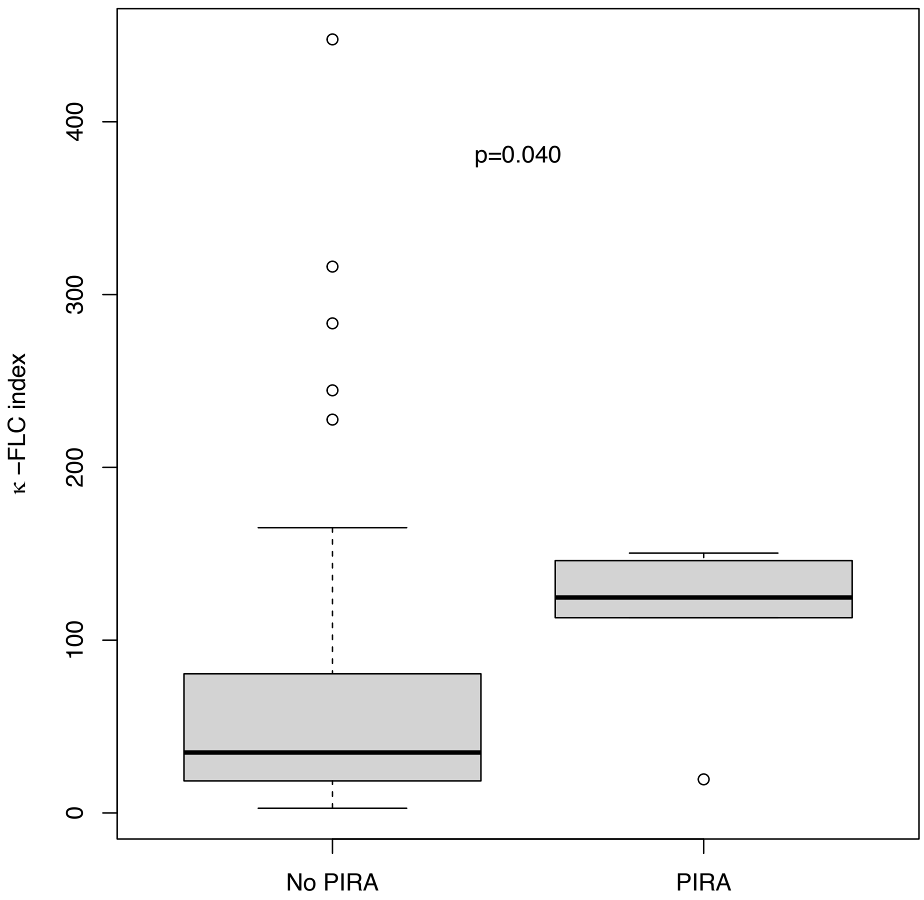

κ-FLC = κ-free light chain. PIRA = progression independent of relapse activity
